# Notes and Notices

**Published:** 1890-05

**Authors:** 


### NOTES AND NOTICES.

International Hahnemannian Association—Notice of Meeting.—The next meeting of the I. H. A. will be held at the "Ocean House," Watch Hill, R. I., June 24th, 25th, 26th, 27th. The hotel rates will be $2.50 per day Watch Hill is about four miles across the bay from Stonington, Conn. Stonington is reached from New York City by the Shore Line R. R. or by the "Stonington Line" of steamers which leave New York late in the afternoon; it is reached from Albany via New York City or by the Boston and Albany R. R. via Worcester; from Boston by the Providence division of the Old Colony R. R. From Stonington steamers run to Watch Hill at short intervals during the day.

This meeting should be the largest ever held. It is very necessary now, that we should make our efforts in behalf of pure Homœopathy known by action. When one so-called homœopathist of New York publicly proclaims in the daily papers that "there is not a strictly homœopathic physician in New York City," and another says that "a homœopathic physician is one who belongs to a homœopathic medical society, no matter if he does have recourse to allopathic remedies"—when such things are uttered in the name of Homœopathy, it is high time for the homœopathic purist to protest and to make his protest known to the public.

You can do this by attending this coming meeting, and early notice is hereby given that you may make all necessary arrangements to be present, for it is a duty that you owe not only to yourself but to all other practitioners of the Homœopathy of Hahnemann. S. A. Kimball, Secretary I. H. A., 124 Commonwealth Ave., Boston, Mass., March 21st, 1890.

An Appeal from the Bureau of Clinical Medicine to Every Member of the International Hahnemannian Association.—*My Dear Doctor:* The Bureau of Clinical Medicine wants something from you. The chairmen of the other bureaus selected their members almost before I was appointed, consequently, and with the sanction of our president, I shall take the "field," and with your help get together the most important collection of clinical material ever presented to any homœopathic society. I am intentionally late in sending this *appeal*, as it has left you free to send *your opinions* to the other bureaus, and now I beg you to send the Bureau of Clinical Medicine *the solid facts.*

Every member can send from five to a hundred valuable clinical observations. Since you began practice, and even since the last, and very valuable, meeting of the society, you have had your heart leap with joy many a time at the wonderful and indisputable effect of the remedy you had given. *Just these facts are what we want in this bureau.* These positive and beautifully illustrative proofs that *Similia similibus curantur* is the law of cure. Confirmations of previous observations are of very great value, especially on the newer remedies. Whether they are short and direct like Hering's characteristics, or as long as the "moral law," makes no difference. Send what most interests you, and what you feel to be so true and valuable that every true homœopath in the land should know you have observed or confirmed it. Remember, the *truth can be often repeated.* Much, very much, is lost to us by members who think every one knows, or should know, what is an every-day fact to them. Now then, every-day facts are what we want, as well as Sunday facts.

As it is especially desirable that all the papers of the bureau should be correct, will you kindly send them so clearly written that no mistake on the part of the printer will be possible.

Upon a postal card I beg you will inform me whether you can support this bureau or not, and if so, when I may look for your papers. Respectfully yours, T. M. Dillingham, Chairman Bureau of Clinical Medicine, 46 West 36th St., New York.

Bureau of Surgery of I. H. A.—In the March number, page 139, we published a communication from Dr. T. Dwight Stow, giving the list of members of the Bureau of Surgery of the I. H. A. Unfortunately two important names—Drs. Carr and Brownell—were left out.

The corrected list is as follows:

Jas. B. Bell, M. D., 178 Commonwealth Ave., Boston.

W. G. Brownell, M. D., Rochester, N. Y.

Allen B. Carr, M. D., Rochester, N. Y.

Prof. E. Carleton, M. D., 53 W. 45th St., New York City.

Thos. M. Dillingham, M. D., 46 W. 36th St., New York City.

A. McNeil, M. D., 220 Turk St., San Francisco.

Miss E. J. Myers, M. D., 302 West 12th St., New York City.

Homœopathic Medical College of Missouri.—The thirty-first annual commencement exercises were held on the evening of March 13th, 1890, at Pickwick Theatre, and the house was crowded even to the galleries.

The programme was excellent. The Rev. J. D. Wilson, D. D., delivered a very eloquent opening prayer, and the Rev. J. R. Ford, D. D., addressed the class on part of the Faculty in a masterly manner, his advice being sound and practical, yet put in such a kindly, earnest way that it was deeply felt and appreciated.

The awarding of the prizes and presentation of flowers was made by Prof. I. D. Foulon, A. M., M. D., LL. B., in his inimitable way. At one moment the audience was convulsed with laughter, and the next all but moved to tears.

Dr. W. A. Edmonds, A. M., M. D., conferred the degree of M. D. upon the class in a well-timed and dignified address, there being twenty-three regular graduates, one ad eundem and one honorary degree. The graduates were as follows:

Max Aszman, W. E. Bruce, J. H. Callan, T. J. Haughton, L. H. Lemke, F. E. Gladwin, L. E. Schoch, Frank Kirsh, F. H. Aufderheide, C. A. Brown, C. A. Canfield, A. E. Knieburg, M. E. McCarty, A. C. Goodbar, E. M. Santee, R. B. Noe, D. E. Archer, E. A. Bohm, D. M. Gibson, H. C. Irwin, G. H. Moser, C. F. Lee, M. E. Tucker. Ad eundem to Dr. Vogt, and the honorary degree to Prof. Foulon.

The most attractive feature of the programme was the musical part, Mr. Charles Kunkel, Mrs. Mayo-Rhodes, Miss Claire Stephens, and the Amphion Quartette—all artists in their specialties—appearing at their best.

The Southern Journal of Homœopathy has again changed hand The former proprietor, Dr. G. G. Clifford, of San Antonio, Texas, has sold the entire property to Mr. T. Engelbach, the well-known Homœopathic pharmacist of 150 Canal St., New Orleans, from which address the *Journal* will hereafter be issued.

Dr. C. E. Fisher, of San Antonio, Texas, the former editor, once more takes the post of editor. All business communications must be sent to Mr. Engelbach. All exchanges, books for review, and contributions to its pages must be sent to Dr. Fisher, of San Antonio.

